# In This Issue

**Published:** 2010

## INTEGRATING HIV/AIDS AND ALCOHOL RESEARCH

Most people who are at risk for HIV infection also consume alcohol. Alcohol use and misuse play an important role in the acquisition, progression, and transmission of HIV, as well as in treatment adherence. According to Drs. Kendall J. Bryant, Steve Nelson, R. Scott Braithwaite, and Deidra Roach, it is crucial to bring the two fields of alcohol and HIV research together and to integrate behavioral and biological research to identify strategies to prevent the spread of HIV infection in alcohol-abusing populations. Equally important are efforts to improve the often-ineffective treatment of patients with HIV/AIDS and alcohol problems and to strengthen translational research in order to effectively implement promising approaches on a large scale. (pp. 167–178)

## HIV RISK AND THE ALCOHOL ENVIRONMENT: ADVANCING AN ECOLOGICAL EPIDEMIOLOGY FOR HIV/AIDS

Societal structures, neighborhoods, and social contexts are important factors influencing the risk of contracting HIV/AIDS. This article by Drs. Richard Scribner, Katherine P. Theall, Neal Simonsen, and William Robinson explores the need for an ecological epidemiological approach to studying HIV/AIDS. This approach is built around a socio-ecological framework, including influences at the individual level, the interpersonal level, the neighborhood level, and the societal level. This framework provides the basis for a conceptual model with specific risk factors at each of these levels and cross-level associations. (pp. 179–183)

## SOCIAL AND STRUCTURAL HIV PREVENTION IN ALCOHOL-SERVING ESTABLISHMENTS: REVIEW OF INTERNATIONAL INTERVENTIONS ACROSS POPULATIONS

Alcohol consumption is associated with increased risk for sexually transmitted infections (STIs), including HIV/AIDS. This article by Dr. Seth C. Kalichman reviews HIV prevention interventions, especially multilevel interventions, conducted in bars, taverns, and informal drinking venues. Interventions designed to reduce HIV risk by altering the social interactions within drinking environments have demonstrated mixed results. Nevertheless, a small number of studies that looked at multilevel approaches as a way of intervening at both social and structural levels have shown encouraging results (pp. 184–194).

## BIOMEDICAL APPROACHES TO HIV PREVENTION

Approximately 80 percent of the more than 60 million people infected with HIV since the epidemic was first detected were infected via sexual intercourse. This article by Drs. Kenneth H. Mayer, Margie Skeer, and Matthew J. Mimiaga describes a variety of biomedical approaches to HIV prevention that have been evaluated or which currently are being studied. These approaches include an anti-HIV vaccine; topical protection treatments; additional biomedical and barrier approaches, such as controlling sexually transmitted diseases, male circumcision, and diaphragm use; and substance abuse treatment. The article also reviews the use of topical versus oral antiretrovirals to prevent HIV transmission, antiretroviral treatment as prevention, and the role of alcohol and other drug use in HIV prevention. (pp. 195–202)

## ALCOHOL’S ROLE IN HIV TRANSMISSION AND DISEASE PROGRESSION

In studies conducted on rhesus macaques infected with simian immunodeficiency virus (SIV), data suggest that the increased SIV levels observed in alcohol-consuming animals may represent an increase in virus production as opposed to a decrease in host defense. Results also suggest that changes in nutritional balance and metabolism, as a possible consequence of a proinflammatory state, together with increased virus production in animals consuming alcohol, accelerate SIV and possibly HIV disease progression. This article by Drs. Ivona Pandrea, Kyle I. Happel, Angela M. Amedee, Gregory J. Bagby, and Steve Nelson describes how the behavioral, virologic, pharmacologic, and immunologic effects of alcohol combine to result in suboptimal treatment responses and the more rapid development of AIDS. (pp. 203–218)

## HIV/AIDS, COMORBIDITY, AND ALCOHOL: CAN WE MAKE A DIFFERENCE?

Alcohol use is common among people at risk for HIV and has a central modifiable effect on their health outcomes, especially now that those with HIV are living longer with the disease. Alcohol use among people with HIV can affect medication adherence and antiretroviral resistance, as well as increase risky sexual behavior. This article by Drs. Amy Justice, Lynn Sullivan, and David Fiellin reviews the prevalence of alcohol use among people with HIV and the complex and interacting part that alcohol use plays in HIV and selected comorbid diseases. The article also describes ongoing plans for continued longitudinal observation and outlines plans for a multilevel strategy implementation trial within the Veterans Aging Cohort Study (VACS). (pp. 258–266)

## INTERVENTIONS TARGETING HIV-INFECTED RISKY DRINKERS: DROPS IN THE BOTTLE

Excessive drinking within the HIV-infected population is associated with numerous adverse affects; thus, interventions to prevent alcohol misuse in this population are urgently needed. In this article, Drs. Jeffrey H. Samet and Alexander Y. Walley summarize the findings of clinical trials of a range of interventions among HIV-infected people with past or current unhealthy alcohol use and dependence. The populations included in these clinical trials encompass HIV-infected people with past or current unhealthy alcohol use, HIV-infected people of whom at least 10 percent currently use alcohol, and alcohol users at high-risk for HIV infection. Data are limited but does provide some valuable guidance for the development of additional prevention approaches for this vulnerable population. (pp. 267–279)

## INFLUENCE OF ALCOHOL CONSUMPTION ON ADHERENCE TO AND TOXICITY OF ANTIRETROVIRAL THERAPY AND SURVIVAL

Although antiretroviral therapy (ART) has significantly reduced mortality among HIV-infected people, patients must strictly adhere to the treatment regimen to ensure its effectiveness. As Drs. R. Scott Braithwaite and Kendall Bryant explain, alcohol consumption impacts HIV-infected patients through a variety of pathways, some of which are related to ART and its effectiveness. For example, alcohol may exacerbate the hepatotoxicity of the medications used in ART. Even more importantly, alcohol consumption may interfere with patient adherence to ART, thereby compromising treatment effectiveness. The authors summarize the findings of studies demonstrating that even at nonbinge-drinking levels, alcohol consumption can reduce treatment adherence sufficiently to impact the patients’ survival. These observations may have implications for the design of optimal treatment approaches for HIV-infected people who consume alcohol. (pp. 280–287)

## FOCUS ON THE LUNG

Evidence suggests that HIV–1 infection and chronic alcohol abuse adversely affect lung health through multiple mechanisms. People with HIV who consume alcohol are susceptible to pneumonia, particularly pneumonia caused by certain serious pathogens, as well as acute respiratory distress syndrome, a serious acute lung condition; asthma; emphysema; and chronic bronchitis, report Drs. David Quintero and David M. Guidot. The authors review the association between HIV–1 infection, alcohol-consumption, and a number of serious acute and chronic lung diseases. They also explore some of the mechanisms that may underlie these associations and suggest potential new treatments based on these findings. (pp. 219–228)

## FOCUS ON THE LIVER: ALCOHOL USE, HIGHLY ACTIVE ANTIRETROVIAL THERAPY, AND LIVER DISEASE IN HIV-INFECTED PATIENTS

Since the introduction of highly active antiretroviral therapy (HAART), liver disease is emerging as a major cause of morbidity and mortality among HIV-infected patients. In this article, Drs. Shirish Barve, Rama Kapoor, Akshata Moghe, Julio A. Ramirez, John W. Eaton, Leila Gobejishvili, Swati Joshi-Barve, and Craig J. McClain examine the factors that may contribute to the increased risk of liver disease. These factors include HAART hepatotoxicity, coinfection with hepatitis B and C virus, and alcohol abuse. The authors also review potential mechanisms that may contribute to alcohol- and HIV/HAART-mediated hepatotoxicity, including dysregulation of signaling molecules (i.e., cytokines) and dysfunction of cell components (i.e., proteasomes and mitochondria). (pp. 229–236)

## FOCUS ON THE HEART: ALCOHOL CONSUMPTION, HIV INFECTION, AND CARDIOVASCULAR DISEASE

New and more effective antiretroviral therapies, such as combination antiretroviral therapy, have increased the lifespan of people infected with HIV. As a result of longer life expectancies, chronic conditions such as cardiovascular disease (CVD) also have increased in prevalence. As in noninfected people, alcohol use and abuse in HIV-infected people may influence their risk of CVD. This article by Drs. Matthew S. Freiberg and Kevin L. Kraemer explores the relationships between HIV infection, alcohol use, and CVD. Drs. Freiberg and Kraemer also describe how excessive alcohol consumption may work synergistically with HIV to promote the development of CVD. (pp. 237–246)

## FOCUS ON THE BRAIN: HIV INFECTION AND ALCOHOLISM— COMORBIDITY EFFECTS ON BRAIN STRUCTURE AND FUNCTION

The combined effects of alcoholism and HIV infection on the brain’s structure, chemistry, and function are devastating, and it is suggested that the damage is particularly prominent in patients whose HIV infection has progressed to AIDS. In this article, Ms. Margaret J. Rosenbloom, Drs. Edith V. Sullivan, and Adolf Pfefferbaum summarize the findings of neuroimaging studies using noninvasive magnetic resonance (MR) technologies for in vivo examination of brain structure (i.e., conventional magnetic resonance imaging [MRI]), white matter fiber integrity (i.e., MR diffusion tensor imaging [DTI]), and brain chemistry (i.e., MR spectroscopy [MRS]) in people with both alcoholism and HIV infection compared with single-diagnosis groups and unaffected control subjects. These studies support the hypothesis that alcoholism is a major risk factor for the development of brain abnormalities in people with HIV infection. (pp. 247–257)

